# Evaluation of the sensitivity and specificity of three diagnostic tests for *Coxiella burnetii* infection in cattle and buffaloes in Punjab (India) using Bayesian latent class analysis

**DOI:** 10.1371/journal.pone.0254303

**Published:** 2022-05-05

**Authors:** Eleftherios Meletis, Ravikiran Keshavamurthy, Balbir Bagicha Singh Dhaliwal, Rabinder Singh Aulakh, Navneet Dhand, Polychronis Kostoulas

**Affiliations:** 1 Faculty of Public and One Health, School of Health Sciences, University of Thessaly, Karditsa, Greece; 2 School of Public Health & Zoonoses, Guru Angad Dev Veterinary and Animal Sciences University, Ludhiana, Punjab, India; 3 Paul G. Allen School for Global Health, Washington State University, Pullman, Washington, United States of America; 4 Sydney School of Veterinary Science, The University of Sydney, Camden, New South Wales, Australia; University of Lincoln, UNITED KINGDOM

## Abstract

Q Fever is a zoonotic disease of significant animal and public health concern, caused by *Coxiella burnetii (C*. *burnetii)*, an obligate intracellular bacterium. This study was done to evaluate the diagnostic sensitivity (D*Se*) and diagnostic specificity (D*Sp*) of three diagnostic methods to diagnose *C*. *burnetii* infection in cattle and buffaloes in Punjab, India: an indirect ELISA method applied in serum samples and a trans-Polymerase Chain Reaction (trans-PCR) technique applied in milk samples and genital swabs, using a Bayesian latent class analysis. Conditional independence was assumed between the tests, given (i) the different biological principle of ELISA and trans-PCR and (ii) the fact that the trans-PCR was performed on different tissues. The ELISA method in the serum samples showed the highest D*Se* of 0.97 (95% Probability Intervals (PIs): 0.93; 0.99) compared to the trans-PCR method applied in milk samples 0.76 (0.63; 0.87) and genital swabs 0.73 (0.58; 0.85). The D*Sps* of all tests were high, with trans-PCR in genital swabs recording the highest D*Sp* of 0.99 (0.98; 1), while the D*Sp* of trans-PCR in milk samples and ELISA in serum samples were 0.97 (0.95; 0.99) and 0.95 (0.93; 0.97) respectively. The study results show that none of the applied tests are perfect, therefore, a testing regimen based on the diagnostic characteristic of the tests may be considered for diagnosis of *C*. *burnetii*.

## Introduction

Q Fever is a zoonotic disease that was first described by Edward Holbrook Derrick [1] in Queensland. Q fever cases have been reported worldwide, except in New Zealand and Antarctica [2, 3] and according to the OIE Terrestrial Animal Health Code, OIE countries and territories are obligated to report occurrences of the disease [4]. Q stands for “query” and this designation was applied when the causative agent of the disease was unknown [4].

*Coxiella burnetii (C*. *burnetii)*, an obligate intracellular Gram-negative coccobacillary bacterium was identified as the causative agent of Q fever in 1938 [5, 6]. *C*. *burnetii*, as a Gram-negative bacterium can display two different phenotypes. Phase I bacteria are highly virulent, while Phase II bacteria are avirulent [7]. *C*. *burnetii*, has been isolated from many domestic and wild animals, birds and arthropods; however, cattle, buffaloes, sheep, goats and humans are commonly affected [8]. Further, many Ixodidae and Argasidae ticks are considered reservoirs of the bacterium [9].

Circulation of the bacterium has been described in wild animals’ populations and arthropods and dispersion of the bacterium in domestic populations can occur through air, direct contact and animal secretions/excretions (e.g., vaginal discharge, placenta, milk, feces, urine, saliva, amniotic fluid) [10].

Limited information on the pathogenesis of *C*. *burnetii* in domestic animals is available, while under laboratory conditions several studies based on different animal models (e.g., guinea pigs, mice) have been conducted [7]. In most cases, inhalation of aerosols or dust contaminated with birth fluids is described as the main route of infection [11]. The pathogenesis and associated histopathological findings depend on the route of infection [7]. Large *C*. *burnetii* concentrations are present in the infected placenta and amniotic fluid, while infected cows can shed the microorganism in milk for up to 32 months [12]. Guatteo et al. (2006) [13] studied three different shedding routes—milk, vaginal mucus, feces—of the bacterium. Study results indicated no predominant *C*. *burnetii* shedding route and for the majority of shedder cows one shedding route was identified [13]. Therefore, testing protocols aiming to detect *C*. *burnetii* in more than one shedding route should be preferred for detection of infection in ruminants.

In livestock, the disease is usually subclinical, but the clinical form is associated with reproductive complications such as abortions, stillbirth, weak calves, repeat breeding and general clinical signs (e.g., anorexia) [14–16]. Further, *C*. *burnetii* excretion via feces, vaginal mucus and milk has been reported, sometimes independent of an abortion history [7].

The host’s immune response to limit the infection has been studied in animal models. Macrophages during infection are the major target cells. Furthermore, T-cells -associated with cellular immunity- are critical for *C*. *burnetii* clearance after infection. On the other hand, B-cells -associated with humoral immunity- are important for tissue damage prevention. Antibody detection differs between the two Phases and the species, therefore serological testing requires the ability to detect antibodies against both phase I and phase II antigen. Typically, antibodies can be detected as early as 14 days post-inoculation for anti-C. burnetii phase II antibodies and 21 days in the case of anti-C. burnetii phase I antibodies [7].

In humans, the disease is observed in the (i) acute form, where flu-like symptoms, atypical pneumonia, hepatitis and cardiac involvement may be present and (ii) chronic form, that is more severe, than the acute, and fatal without appropriate therapy [17]. Therefore, *C*. *burnetii* is characterized as a microorganism of great animal and public health concern.

Since there are no pathognomonic characteristics associated with *C*. *burnetii* infection, diagnosis poses a challenge. Many diagnostic tests, that are based on either the detection of the immune response of the host e.g., ELISA detects antibodies directed against *C*. *burnetii* or the microorganism like Polymerase Chain Reaction (PCR) that detects bacterial DNA, have been used in epidemiological studies for *C*. *burnetii* to obtain estimates of the incidence and prevalence [18]. However, prevalence estimation depends on the test’s diagnostic sensitivity *(DSe)* and diagnostic specificity *(DSp)*, therefore, accurate diagnostic accuracy measures are important.

*C*. *burnetii* infection was first described in India in 1952 in a cattle herd and the first human case was reported in 1953 [19]. Since then, the disease has been reported in several studies in India and an increasing trend in prevalence is reported, outlining *C*. *burnetii* as a potential threat to public health [16, 20–25]. Reported prevalence estimates from studies in India, conducted in a frequentist framework, assume that the applied diagnostic tests have perfect *DSe* and *DSp* or imperfect, but known, measures of test accuracy [22].

Livestock production plays an important role and is directly associated with the health and financial progress of rural households in India. According to 20^th^ Indian livestock census conducted in 2019, 36% (192.5 million) and 20.5% (109.85 million) of the total livestock (535.78 million) is contributed by cattle and buffaloes, respectively [26]. The average herd size in India is comparatively low and most of the livestock producers own less than 5 animals [27–29]. Punjab is an agrarian state located in the Northern India (Latitude of 30°4’N and Longitude 75° 5’ E) with a cattle and buffalo population of 2.5 and 4 million, respectively [26]. Cattle and buffalo are the most important livestock species reared in the state.

Endemic and emerging zoonotic diseases are responsible for huge economic losses in the livestock production. Further zoonotic diseases pose a great challenge to public health sector in India. Q Fever in India still is a neglected zoonotic disease that lacks appropriate attention, strategies for detection and prevention due to lack of epidemiological data and diagnostics, poor disease surveillance, and lack of disease awareness even among the public health professionals [25, 30].

Further, for the species under investigation in this study—cattle and buffaloes- the course of infection is considered similar [18].

Objective of this study is the diagnostic evaluation of the applied tests to detect *C*. *burnetii* infection in cattle and buffalo animals in Punjab. The acquired results will enable animal health authorities towards making appropriate policy decisions in controlling the disease.

Since no gold standard is reported for *C*. *burnetii*, the study was conducted in a Bayesian framework, following the STARD-BLCM guidelines [31]. The STARD-BLCM checklist is available as a supplementary material (S1 Appendix).

## Materials and methods

### Ethics approval

This study was approved by the Institutional Animal Ethical Committee, Guru Angad Dev Veterinary and Animal Sciences University (GADVASU/2017/IAEC/42/02).

### Study design

The study design is presented in detail elsewhere [16]. Briefly, a multi-stage sampling design was performed. Twenty-two villages, one per district, of the Punjab state were selected. The family households keeping cattle and/or buffalo herds were considered as eligible for this study. The number of households sampled in each village was selected proportional to the number of households keeping cattle and/or buffalo herds in a village. Overall, 179 households (dairy cattle or buffalo herds) participated in the study. We worked towards collecting samples from all the animals for each household. However, many farmers were reluctant to provide samples from some of their animals. A consent of the livestock owners was obtained before the collection of samples. The sampled proportion of all eligible households and animals was 72.5% and 53.4%, respectively [16].

The sampling unit in this study was cattle and buffaloes reared for milk. In the analysis cattle and buffaloes were considered as one population, referred as domestic bovine population, because (i) both species are members of Bovidae family [32] and (ii) the course of the *C*. *burnetii* infection is similar in both species [18]. Under this setting, the target population of the study was the domestic bovine population in the Punjab state.

### Sample collection

Blood and genital swab samples were collected from the selected cattle and buffaloes. In addition, milk samples, from lactating female animals, were collected. Blood samples were collected aseptically from the jugular vein. Puncture area was cleaned with 70% alcohol and venipuncture was done using a fresh needle. Approximately 5 ml of blood was withdrawn from each animal in a sterile vacutainer. For the molecular study, genital swabs were collected from both male and female animals using sterile cotton swabs. Vaginal swab samples from female animals were collected by carefully inserting the swab into the vaginal cavity about 10 cm through followed by gently rotating the swab. In males, preputial swabs were collected by swabbing the penile and preputial surface. For the collection of milk samples from the lactating animals, udder and teats were cleaned using germicidal teat dip and three to four streams of milk was discarded before sampling to minimize risk of sample contamination. About 15 ml of milk sample was collected using sterile screw-capped vials.

All samples were transported to the laboratory on the sampling day and stored at -20°C. The sera were separated within 24h in the sterile cryovials before storing at -20°C until screened [16].

Overall, 610 blood samples, 610 genital swabs and 361 milk samples were collected from the study population. Therefore, the samples were split into two subpopulations; subpopulation-1 (subp_1) includes lactating female animals (n = 361) and subpopulation-2 (subp_2) includes male and non-lactating female animals (n = 249). Overall, 221 of the sampled animals (36.2.%) were pregnant.

### Laboratory tests

The 361 milk samples and 610 genital swabs were screened with PCR to detect bacterial DNA, Briefly, using DNeasy Blood and Tissue kit (Qiagen, Maryland, USA). Specifically, a trans-PCR assay was used to detect *C*. *burnetii* particles based on two transposon-like repetitive regions of the microorganism, called IS1111 transposase gene [33] using a set of published primers; Trans1: 5’-TAT GTA TCC ACC GTA GCC AGT C-3’ and Trans2: 5’-CCC AAC AAC ACC TCC TTA TTC-3’ targeting 687 bp of IS1111a insertion sequence of *C*. *burnetii* [34].

The 610 serum samples were screened for *C*. *burnetii* IgG antibodies using the commercial Q Fever indirect ELISA kit with Phase I and Phase II (ELISA Kit for serodiagnosis of Q Fever in cattle and small ruminants, Monowell, Bio-X Diagnostics, Rochefort, Belgique). The two Phases capture both the acute and chronic infection form. In particular, IgG antibody titers against Phase I antigens are elevated during the acute phase, whereas IgG antibody titers against Phase I and Phase II are elevated during the chronic phase (ELISA Kit for serodiagnosis of Q Fever in cattle and small ruminants, Monowell, Bio-X Diagnostics, Rochefort, Belgique). ELISA quantifies the immune response of the host against *C*. *burnetii* and does not provide information about the presence or absence of the bacterium. Broadly, serological techniques are useful for screening purposes e.g., monitor the vaccination effectiveness or detecting previous/recent natural infection. This is not applicable in India, because a vaccination program for *C*. *burnetii* does not exist [25].

### Bayesian latent class analysis

The diagnostic accuracy of the applied tests was estimated using a Bayesian latent class model (BLCM). Traditionally, in the absence of a “gold standard”, latent class models [35] can be used to obtain unbiased estimates. Over the last decades, Bayesian framework has been applied in latent class analyses, due to their flexibility, incorporation of prior knowledge and software availability [36, 37]. To ensure transparency and extrapolation of the study results, the STARD-BLCM reporting guidelines for diagnostic accuracy studies that use BLCMs were followed [31].

#### Definition of infection status

Explicit description of the biological principle of each applied test is crucial towards the structure of any BLCM model. Latent variables are hidden, or unknown and probabilistic estimates can be made for them in conjunction with what the tests actually detect [38]. Specifically, the applied PCR technique in the milk samples (PCR-Milk) and the genital swabs (PCR-Genital) detect bacterial DNA i.e., presence or absence of the *C*. *burnetii* microorganism and ELISA measures antibodies titers i.e., immune host response (IgG ELISA). In this study, the latent variable is the true infection status and is defined as any condition where the bacterium has entered the organism and persisted long enough to produce a detectable immune response in the host at any time during their life.

Estimations were based on the cross-classified results (Table 1) of the applied tests in the two subpopulations described above.

**Table 1:** Cross-classified results of IgG ELISA, PCR-Genital and PCR-Milk.

Sub-population	IgG ELISA^b^	PCR-Genital^a^	PCR-Milk^c^	Total
Lactating female animals	+	+	+	1
	+	+	-	0
	+	-	+	3
	+	-	-	23
	-	+	+	0
	-	+	-	3
	-	-	+	7
	-	-	-	324
	Total			361
Non-lactating female animals + Male animals	+	+	NA^d^	0
	+	-	NA	6
	-	+	NA	0
	-	-	NA	243
	Total			249

^a^PCR-Genital: Polymerase Chain Reaction (PCR) in genital swabs.

^b^PCR-Milk: PCR in milk samples.

^c^IgG ELISA: ELISA in serum samples.

^d^NA: Not performed.

#### Model assumptions

BLCM models, in the absence of a gold standard, for *DSe* and *DSp* estimation can be constructed under different assumptions. An applied set of assumptions adopted by Hui and Walter model (two tests—two populations) state that (i) the population is divided into two or more subpopulations in which two or more tests are evaluated (ii) *DSe* and *DSp* of each test remain constant across both species and both subpopulations and (iii) all tests are conditionally independent given infection status [39]. According to the literature, previous Bayesian latent-class analyses for *C*. *burnetii* infection indicate that the *DSe* and *DSp* of ELISA do not vary between species [18, 40]. Conditional independence can be assumed, on the basis that ELISA and PCR do not measure similar biological processes [41]. Also, PCR-Milk and PCR-Genital were applied to different organs, therefore, presence (absence) of the infectious agent to one organ does not imply presence (absence) to other organs. Even though, the existence of distinct difference of the true prevalence between the subpopulations is proven to influence the precision of the estimates; this is not applicable in this case, i.e., the two subpopulations have the same true prevalence [39].

#### Model description

Bayesian modelling extracts the posterior probability given prior information and the likelihood function. The likelihood is computed through a statistical model for the observed data.

The models for the two subpopulations were structured assuming that the various test combinations follow the multinomial distribution. Specifically,

y_subp_1[1:Q,1:Q,1:Q]~dmulti(p1[1:Q,1:Q,1:Q],n1)

y_subp_2[1:Q,1:Q]~dmulti(p2[1:Q,1:Q],n2)

where y_subp_1 and y_subp_2 are the counts of various test combinations, Q = {1,2} the dichotomized test result i.e., 1 for positive and 2 for negative, n1 & n2 the two population sizes and p1 & p2 the probabilities of observing each test combination.

Based on this notation the frequencies of possible test outcomes can be calculated as followed:

$$p1[1,1,1]=pi\;*\;{Se}_{ELISA}\;*\;{Se}_{Genital}^{PCR}\;*\;{Se}_{Milk}^{PCR}\;+\;(1\;-\;pi)\;*\;({1\;-\;Sp}_{ELISA})\;*\;(1\;-\;{Sp}_{Genital}^{PCR})\;*\;(1\;-\;{Sp}_{Milk}^{PCR})$$

$$p1[1,1,2]=pi\;*\;{Se}_{ELISA}\;*\;{Se}_{Genital}^{PCR}\;*\;(1\;-\;{Se}_{Milk}^{PCR})\;+\;(1\;-\;pi)\;*\;({1\;-\;Sp}_{ELISA})\;*\;(1\;-\;{Sp}_{Genital}^{PCR})\;*\;{Sp}_{Milk}^{PCR}$$

$$p2[1,1]=pi\;*\;{Se}_{ELISA}\;*\;{Se}_{Genital}^{PCR}+\;(1\;-\;pi)\;*\;({1\;-\;Sp}_{ELISA})\;*\;(1\;-\;{Sp}_{Genital}^{PCR})$$

$$p2[2,2]=pi\;*\;(1\;-\;{Se}_{ELISA})\;*\;(1\;-\;{Se}_{Genital}^{PCR})+\;(1\;-\;pi)\;*\;{Sp}_{ELISA}\;*\;{Sp}_{Genital}^{PCR}$$

Under this setting, the parameters to be estimated are seven (i.e., Se_ELISA_, Se_PCR-Genital_, Se_PCR-Milk_, Sp_ELISA_, Sp_PCR-Genital_, Sp_PCR-Milk_ and pi (true prevalence)), while the degrees of freedom offered by the data are seven. Therefore, identifiability criteria are met and an uniform, noninformative, Beta prior distribution *Be (1*,*1)* can be adopted for all parameters of interest. However, degrees of freedom being higher than or equal to the number of parameters to be estimated is a necessary but not always sufficient condition to ensure identifiability. In this analysis, due to the sparsity of the observed data (i.e., zero cell observations for some of the cross-classified results—see Table 1) the ability of the model to estimate the associated parameters diminishes. The only solution to this problem is to incorporate external information by informing the prior distribution [42]. Hence, informative priors were introduced.

Prior information for the test characteristics was supplied by one of the co-authors (BBSD), an epidemiologist, expert on zoonoses and co-leader of a national project on “Epidemiology, burden and control of zoonotic diseases in India”. Generally, not much is known about the differences in the D*Se* and D*Sp* of similar diagnostic tests in cattle and buffalo populations. In detail, estimates of the mean and the 95^th^ percentile for the DSe and DSp for the three diagnostic methods were provided. The provided details agreed with the literature on diagnostic test evaluation for detecting *C*. *burnetii* infection on cattle and buffaloes [43, 44]. Informative prior Beta distributions were calculated using the PriorGen R Package [45] (Table 2).

**Table 2:** Mean and 95^th^ percentiles for the diagnostic sensitivity (*DSe*) and diagnostic specificity (*DSp*) priors of PCR and ELISA and the corresponding Beta distributions, *Be(a*, *b)*. The prior information was provided by one of the co-authors (BBSD).

Test	Parameter	Mean (%)	95^th^ percentile (%)	Be (a, b)
PCR	*DSe* ^a^	75	85	*Be(32*.*58*, *10*.*86)*
	*DSp* ^b^	95	99	*Be(40*.*78*, *2*.*15)*
ELISA	*DSe*	97	99	*Be(122*.*51*, *3*.*79)*
	*DSp*	90	95	*Be(11*.*74*, *1*.*3)*

^a^Diagnostic Sensitivity.

^b^Diagnostic Spevificity.

#### Markov Chain Monte Carlo convergence and software

Models were run in the freeware program OpenBUGS [46]. Parameter estimates were based on analytical summaries of 100,000 iterations of two chains after a burn-in phase of 5000 iterations. The tools described in [47], were monitored to ensure occurrence of convergence. Specifically, the time series plots for all parameters indicated that the two chains have converged, plus autocorrelations dropped fast. The OpenBUGS code for the final model is available as a supplementary material (S2 Appendix).

#### Sensitivity analysis

The influence of the data and the priors to the posterior estimates was examined by running the same model without informative prior values (i) for all parameters and (ii) for the DSp of the three methods only. A flat noninformative prior distribution for the DSe of the three tests cannot be adopted, because there are very few positive to all two tests animals. Therefore, the DSe is an under-identified parameter, without using informative prior distributions; that is the main limitation of this study.

## Results

The posterior medians and 95% probability intervals (PIs) for the *DSe* and *DSp* of each diagnostic test are summarized in Table 3.

**Table 3:** Posterior medians and 95% probability intervals (PrIs) for the *DSe* and D*Sp* of each diagnostic test using informative Beta prior distributions for the *DSe* and *DSp* of each diagnostic test described in Table 2.

Test	Parameter	Posterior medians and 95% PrIs
IgG ELISA	*DSe*	0.97 (0.93; 0.99)
	*DSp*	0.95 (0.93; 0.97)
PCR-Genital	*DSe*	0.73 (0.58; 0.85)
	*DSp*	0.99 (0.98; 1)
PCR-Milk	*DSe*	0.76 (0.63; 0.87)
	*DSp*	0.97 (0.95; 0.99)

IgG ELISA showed the highest *DSe* with median 0.97 (95% PIs: 0.93; 0.99) compared to PCR-Milk 0.76 (0.63; 0.87) and PCR-genital 0.73 (0.58; 0.85). The *DSps* of all tests were high, with PCR-Genital recording the highest *DSp* median of 0.99 (0.98; 1), while the *DSp* of PCR-Milk and IgG ELISA were 0.97 (0.95; 0.99) and 0.95 (0.93; 0.97) respectively.

### Sensitivity analysis results

The acquired estimates without informative prior distributions (i) for all the parameters and (ii) for the *DSps* of the tests are shown in S1 and S2 Tables. The posterior estimates for the *DSps* are similar in both scenarios. On the other hand, having uniform noninformative priors for all parameters, resulted in lower median posterior estimates and wider 95% PrIs for the DSes; the *DSe* of IgG ELISA was 0.63 (0.17; 0.98), PCR-Genital 0.18 (0.02; 0.77) and PCR-Milk was 0.6 (0.13; 0.98). The posterior estimates for *DSes*, under uniform noninformative priors for the *DSps*, were similar to those reported in Table 3.

## Discussion

In this study, BLCMs were used to estimate the *DSe* and *DSp* of a trans-PCR applied in genital swabs and milk samples to detect *C*. *burnetii* DNA and an ELISA applied in serum samples that detects antibodies against *C*. *burnetii*, in cattle and buffaloes in Punjab, India. BLCMs account for the absence of a gold standard and allow parameter estimation merging two components (i) model structure, based on the observed data and (ii) incorporation of prior information [31].

The study results show that all three tests are highly specific, with PCR-Genital yielding the higher *DSp* [0.99 (0.98; 1)], followed by PCR-Milk [0.97 (0.95; 0.99)] and IgG ELISA [0.95 (0.93; 0.97)]. On the other hand, IgG ELISA has the highest *DSe* [0.97 (0.93; 0.99)], followed by PCR-Milk [0.76 (0.63; 0.87)] and PCR-Genital [0.73 (0.58; 0.85)]. Overall, the posterior medians and 95% PIs for the PCR-Milk and PCR-Genital are comparable, indicating that both tests have the same diagnostic accuracy. Further, the *DSps* for these two tests, are not “prior-driven/dependent”, since both under informative and uniform, noninformative priors for the *DSps* the posterior estimates are approximately the same. However, the reported estimates for the *DSes* of PCR-Milk, and especially PCR-Genital, seem to differ under noninformative and informative prior distributions [informative prior distributions; PCR-Milk 0.76 (0.63; 0.87) and PCR-Genital 0.73 (0.58; 0.85); noninformative prior distributions; PCR-Milk 0.6 (0.13; 0.98) and PCR-Genital 0.18 (0.02; 0.77)]. This makes sense and is due to the scarcity of the data i.e., small number of animals both positive to PCR-Milk and PCR-Genital; the *DSes* under-identified parameters [42]. The reported medians and 95% PIs for the *DSe* of PCR-Milk and PCR-Genital, under uniform noninformative prior distributions are lower and wider. Implementation of informative prior distributions allows shrinkage of the 95% PI for the *DSes*. Therefore, the final *DSe* estimates for the two PCRs can be considered prior-driven; if not prior-dependent. The same holds for IgG-ELISA where even though a high *DSp* is recorded, the *DSe* is “prior-driven/dependent”. The data supply very information, since only thirty-three animals were tested positive in IgG-ELISA [1 PCR-Milk+, PCR-Genital+, 3 PCR Milk+, PCR-Genital-, 23 PCR-Milk-, PCR-Genital-, 6 PCR-Genital-]. Therefore, IgG ELISA *DSe* posterior estimates using uniform, noninformative prior distribution cannot be considered reliable.

Studies on validation of diagnostic tests for *C*. *burnetii* infection using BLCMs have been conducted in cattle, goats, sheep etc. [40, 43, 48]. The reported *DSe* and *DSp* of the tests in our study are comparable between studies and similar between different species e.g., sheep and goats [49]. The posterior medians and 95% PIs for the diagnostic characteristics of IgG-ELISA are similar to those reported in the literature and comparable with the estimates provided by the commercial ELISA kits manufacturers used for detection of antibodies against *C*. *burnetii* in serum samples from cattle [40, 50]. The PCR method applied in milk samples has been evaluated in cattle [44], in a Bayesian framework. The results from our study are similar with the ones reported in [44]. The PCR method applied in genital swabs in cattle has not been evaluated; instead, PCR has been used for bacterial DNA detection in the farm environment [44]. On the other hand, PCR-Genital and PCR-Milk has been described in the sheep and goats [49]. The *DSp* for both PCRs and for the *DSe* of PCR-Genital are similar, while the reported median and 95% PI for the *DSe* of PCR-Milk in [49] is lower [0.42 (0.32; 0.59)].

Conditional independence between PCR-Milk and PCR-Genital was assumed, since, the method was applied to different organs, even though it is based on the same biological principle. Further, primary shedding route has not been identified for *C*. *burnetii* and isolation of the bacterium from more than two organs is rare (Table 1) [13], i.e., presence (absence) of the infectious agent in the genital tract does not imply presence (absence) to milk. The conditional independence assumption for a PCR method applied in milk and vaginal secretions in sheep and goat samples to detect *C*. *burnetii* has been adopted [49]. Therefore, this assumption can be considered valid. Moreover, the specified model has seven degrees of freedom and seven parameters of interest. If conditional independence was not assumed, then two extra parameters for the covariance terms will be added, and the identifiability criteria will not be met (degrees of freedom higher than or equal to the number of parameters of interest), hence, our model would not converge. Therefore, introducing two covariance terms, accounting for conditional dependence between PCR-Milk & PCR-Genital would result in an unidentifiable model. Even though, in our case we adopt the results using informative prior distributions, we do so, due to the scarcity of the data (zero cell observations for animals positive to IgG-ELISA, PCR-Milk, PCR-Genital). Thus, informative prior distributions are added, instead of noninformative, to overcome the sparsity of the data. As shown in S1 Table the specified model using uniform, noninformative priors converges, but results to 95% PIs with high width.

Furthermore, the course of infection in the two species under investigation was considered similar, hence the diagnostic tests properties were assumed constant across species. In India, risk factor investigation studies present contrasting results in the risk of occurrence of *C*. *burnetii* infection in bovine populations. A recent study conducted in Bihar and Assam states of India reported higher risk of infection for buffaloes than in cattle (28.0% compared to 13.6%, p = 0.042) at the species level [23]. However, only 25 buffaloes, compared to 719 cattle, were included in this study [23]. On the other hand, similar investigations in Punjab reported that cattle (adjusted Odds Ratio 3.37, 95% Confidence interval 1.23–9.20, p = 0.02) were associated with larger odds of *C*. *burnetii* positive animal status than buffaloes [30]. Based on these results, our assumption that the course of infection and disease occurrence does not vary much in cattle and buffaloes at the species level is valid and there are other factors that need further investigation.

## Conclusion

This study was conducted to estimate the *DSe* and *DSp* of three tests used for *C*. *burnetii* detection in Punjab, India. IgG ELISA achieved the highest *DSe*, while PCR-Genital had the highest *DSp*. Using BLCMs, with none of the applied tests showed perfect *DSe* and *DSp*, and therefore, could not be used alone to diagnose *C*. *burnetii* infection in domestic bovine populations. Q Fever in India is a neglected zoonotic disease that lacks strategies for detection and prevention due to lack of epidemiological data and diagnostics. Accurate diagnostic methods for *C*. *burnetii* detection are important to capture the increasing trend in prevalence in India and motivate public health professionals for efficient disease surveillance.

## Supporting information

S1 Table Posterior medians and 95% PrIs for the diagnostic sensitivity (*DSe*) and diagnostic specificity (*DSp*) of each diagnostic test using noninformative Beta prior distributions for all parameters of interest. (DOCX)

S2 Table Posterior medians and 95% PrIs for the diagnostic sensitivity (*DSe)* and diagnostic specificity (*DSp)* of each diagnostic test using noninformative Beta prior distributions for the *DSps* of the three tests. (DOCX)

S1 Appendix STARD-BLCM checklist. (DOCX)

S2 Appendix OpenBUGS code. (DOCX)
