# Supplementary material for: Evaluation of the sensitivity and specificity of three diagnostic tests for Coxiella burnetii infection in cattle and buffaloes in Punjab (India) using Bayesian latent class analysis
Source: PLoS One. 2022 May 5;17(5):e0254303. doi: 10.1371/journal.pone.0254303 (PMC9070919; doi:10.1371/journal.pone.0254303)
Supplement: S1 Table — (DOCX) [file pone.0254303.s001.docx]

**S1 Table. Posterior medians and 95% PrIs for the diagnostic sensitivity (*DSe)* and diagnostic specificity (*DSp)* of each diagnostic test using noninformative Beta prior distributions for all parameters of interest.**

| Test | Parameter | Posterior medians and 95% PrIs |
| --- | --- | --- |
| IgG ELISA^a^ | *DSe* | 0.63 (0.17; 0.98) |
|  | *DSp* | 0.96 (0.93; 0.98) |
| PCR-Genital^b^ | *DSe* | 0.18 (0.02; 0.77) |
|  | *DSp* | 0.99 (0.98; 1) |
| PCR-Milk^c^ | *DSe* | 0.6 (0.13; 0.98) |
|  | *DSp* | 0.98 (0.96; 1) |

^a^IgG ELISA: ELISA in serum samples

^b^PCR-Genital: Polymerase Chain Reaction (PCR) in genital swabs

^c^PCR-Milk: PCR in milk samples
