# Supplementary material for: Evaluation of the sensitivity and specificity of three diagnostic tests for Coxiella burnetii infection in cattle and buffaloes in Punjab (India) using Bayesian latent class analysis
Source: PLoS One. 2022 May 5;17(5):e0254303. doi: 10.1371/journal.pone.0254303 (PMC9070919; doi:10.1371/journal.pone.0254303)
Supplement: S2 Table — (DOCX) [file pone.0254303.s002.docx]

**S2 Table. Posterior medians and 95% PrIs for the diagnostic sensitivity (*DSe)* and diagnostic specificity (*DSp)* of each diagnostic test using noninformative Beta prior distributions for the *DSps* of the three tests**.

| Test | Parameter | Posterior medians and 95% PrIs |
| --- | --- | --- |
| IgG ELISA^a^ | *DSe* | 0.97 (0.93; 0.99) |
|  | *DSp* | 0.95 (0.93; 0.97) |
| PCR-Genital^b^ | *DSe* | 0.73 (0.58; 0.85) |
|  | *DSp* | 0.99 (0.98; 1) |
| PCR-Milk^c^ | *DSe* | 0.76 (0.63; 0.87) |
|  | *DSp* | 0.97 (0.95; 0.99) |

^a^IgG ELISA: ELISA in serum samples

^b^PCR-Genital: Polymerase Chain Reaction (PCR) in genital swabs

^c^PCR-Milk: PCR in milk samples
