# Supplementary material for: Evaluation of the sensitivity and specificity of three diagnostic tests for Coxiella burnetii infection in cattle and buffaloes in Punjab (India) using Bayesian latent class analysis
Source: PLoS One. 2022 May 5;17(5):e0254303. doi: 10.1371/journal.pone.0254303 (PMC9070919; doi:10.1371/journal.pone.0254303)
Supplement: S1 Appendix — (DOCX) [file pone.0254303.s003.docx]

**S1 Appendix. STARD-BLCM Checklist**

|  | **Section & Topic** | **No** | **Item** | **Reported on page #** |
| --- | --- | --- | --- | --- |
|  |  |  |  |  |
|  | **TITLE OR ABSTRACT** |  |  |  |
|  |  | **1** | Identification as a study of diagnostic accuracy, using at least one measure of accuracy (such as sensitivity, specificity, predictive values, or AUC) **and Bayesian latent class models** | **1** |
|  | **ABSTRACT** |  |  |  |
|  |  | **2** | Structured summary of study design, methods, results, and conclusions  (for specific guidance, see STARD for Abstracts) | **2** |
|  | **INTRODUCTION** |  |  |  |
|  |  | **3** | Scientific and clinical background, including the intended use and clinical role of the **tests under evaluation** | **2 - 6** |
|  |  | **4** | Study objectives and hypotheses, **such as estimation of diagnostic accuracy of the tests for a defined purpose through BLCM** | **6** |
|  | **METHODS** |  |  |  |
|  | *Study design* | **5** | Whether data collection was planned before the **tests** were performed (prospective study) or after (retrospective study) | **6 - 7** |
|  | *Participants* | **6** | Eligibility criteria **and description of the source population** | **7** |
|  |  | **7** | On what basis potentially eligible participants were identified  (such as symptoms, results from previous tests, inclusion in registry) | **6 – 8** |
|  |  | **8** | Where and when potentially eligible participants were identified (setting, location and dates) | **6 – 8** |
|  |  | **9** | Whether participants formed a consecutive, random or convenience series | **6 – 8** |
|  | *Test methods* | **10** | **Description of the tests under evaluation**, in sufficient detail to allow replication, **and/or cite references** | **8 – 9** |
|  |  | **11** | Rationale for choosing the **tests under evaluation in relation to their purpose** | **8 – 9** |
|  |  | **12** | Definition of and rationale for test positivity cut-offs or result categories of **the tests under evaluation**, distinguishing pre-specified from exploratory | **8 – 9** |
|  |  | **13** | Whether clinical information was available to the performers or readers of **the tests under evaluation** | **8 – 9** |
|  | *Analysis* | **14a** | **BLCM model** for estimating measures of diagnostic accuracy | **9 – 14** |
|  |  | **14b** | **Definition and rationale of prior information and sensitivity analysis** | **14 & S1 and S2 Tables** |
|  |  | **15** | How indeterminate results **of the tests under evaluation** were handled | **No indeterminate results** |
|  |  | **16** | How missing data **of the tests under evaluation** were handled | **No missing data** |
|  |  | **17** | Any analyses of variability in diagnostic accuracy, distinguishing pre-specified from exploratory | **9 – 13** |
|  |  | **18** | Intended sample size and how it was determined | **7 - 8** |
|  | **RESULTS** |  |  |  |
|  | *Participants* | **19** | Flow of participants, using a diagram | **Not applicable** |
|  |  | **20** | Baseline demographic and clinical characteristics of participants | **7 – 8** |
|  |  | **21** | **The distribution of the targeted conditions is unknown, hence the use of BLCM** | **Not applicable** |
|  |  | **22** | Time interval and any clinical interventions between **the tests under evaluation** | **7 - 8** |
|  | *Test results* | **23** | Cross tabulation of the **tests’ results (or for continuous tests results their distribution by infection stage)** | **Table 1** |
|  |  | **24** | Estimates of diagnostic accuracy **under alternative prior specification** and their precision (such as 95% **credible/probability intervals**) | **14 – 15, Table 3, Supplementary Tables 1-2** |
|  |  | **25** | Any adverse events from performing **the tests under evaluation** | **Not applicable** |
|  | **DISCUSSION** |  |  |  |
|  |  | **26** | Study limitations, including sources of potential bias, statistical uncertainty, and generalisability | **14, 16 – 19** |
|  |  | **27** | Implications for practice, including the intended use and clinical role of **the tests under evaluation in relevant settings (clinical, research, surveillance etc.)** | **16 – 19** |
|  | **OTHER INFORMATION** |  |  |  |
|  |  | **28** | Registration number and name of registry | **Not applicable** |
|  |  | **29** | Where the full study protocol can be accessed | **Not applicable** |
|  |  | **30** | Sources of funding and other support; role of funders | **19** |
|  |  |  |  |  |

STARD-BLCM stands for “Standards for the Reporting of Diagnostic accuracy studies that use Bayesian Latent Class Models” and is a modification of the STARD statement (which was recently updated to STARD2015). STARD-BLCM aims to facilitate improved quality of reporting for diagnostic accuracy studies that use Bayesian latent class models in the absence of a reference standard. The proposed modifications are relevant to both Bayesian and frequentist estimation methods but the focus is on the former.

More information for STARD (STARD2015) can be found at: [http://www.equator-network.org/reporting-guidelines/stard](http://www.equator-network.org/reporting-guidelines/stard/)

More information for STARD-BLCM can be found at: [http://www.equator-network.org/reporting-guidelines/stard-blcm](http://www.equator-network.org/reporting-guidelines/stard-blcm/)
