# Supplementary material for: Evaluation of the sensitivity and specificity of three diagnostic tests for Coxiella burnetii infection in cattle and buffaloes in Punjab (India) using Bayesian latent class analysis
Source: PLoS One. 2022 May 5;17(5):e0254303. doi: 10.1371/journal.pone.0254303 (PMC9070919; doi:10.1371/journal.pone.0254303)
Supplement: S2 Appendix — (DOCX) [file pone.0254303.s004.docx]

**S2 Appendix. OpenBUGS Code**

#model specification

# Animals = Cattle & Buffaloes (i.e. Domestic bovine population)

#sub_population_1 - Lactating female animals

y1[1:Q, 1:Q, 1:Q] ~ dmulti(p1[1:Q, 1:Q, 1:Q], n1)

#sub_population_2 - non-Lactating female & male animals

y2[1:Q, 1:Q] ~ dmulti(p2[1:Q, 1:Q], n2)

#Test 1 - IgG_ELISA

#Test 2 - PCR_Genital

#Test 3 - PCR_Milk

p1[1,1,1] <- pi*Se[1]*Se[2]*Se[3] + (1-pi)*(1-Sp[1])*(1-Sp[2])*(1-Sp[3])

p1[1,1,2] <- pi*Se[1]*Se[2]*(1-Se[3]) + (1-pi)*(1-Sp[1])*(1-Sp[2])*Sp[3]

p1[1,2,1] <- pi*Se[1]*(1-Se[2])*Se[3] + (1-pi)*(1-Sp[1])*Sp[2]*(1-Sp[3])

p1[1,2,2] <- pi*Se[1]*(1-Se[2])*(1-Se[3]) + (1-pi)*(1-Sp[1])*Sp[2]*Sp[3]

p1[2,1,1] <- pi*(1-Se[1])*Se[2]*Se[3] + (1-pi)*Sp[1]*(1-Sp[2])*(1-Sp[3])

p1[2,1,2] <- pi*(1-Se[1])*Se[2]*(1-Se[3]) + (1-pi)*Sp[1]*(1-Sp[2])*Sp[3]

p1[2,2,1] <- pi*(1-Se[1])*(1-Se[2])*Se[3] + (1-pi)*Sp[1]*Sp[2]*(1-Sp[3])

p1[2,2,2] <- pi*(1-Se[1])*(1-Se[2])*(1-Se[3]) + (1-pi)*Sp[1]*Sp[2]*Sp[3]

p2[1,1] <- pi*Se[1]*Se[2] + (1-pi)*(1-Sp[1])*(1-Sp[2])

p2[1,2] <- pi*Se[1]*(1-Se[2]) + (1-pi)*(1-Sp[1])*Sp[2]

p2[2,1] <- pi*(1-Se[1])*Se[2] + (1-pi)*Sp[1]*(1-Sp[2])

p2[2,2] <- pi*(1-Se[1])*(1-Se[2]) + (1-pi)*Sp[1]*Sp[2]

#Uniform - Non-informative prior for prevalence

pi ~ dbeta(1, 1)

#Informative prior information for the three applied tests

# Test 1 - IgG_ELISA

#Se[1] ~ dbeta(122.51, 3.79)

#Sp[1] ~ dbeta(11.74, 1.3)

# Test 2 - PCR_Genital

#Se[2] ~ dbeta(32.58, 10.86)

#Sp[2] ~ dbeta(40.78, 2.15)

#Test 3 - PCR_Milk

#Se[3] ~ dbeta(32.58, 10.86)

#Sp[3] ~ dbeta(40.78, 2.15)
